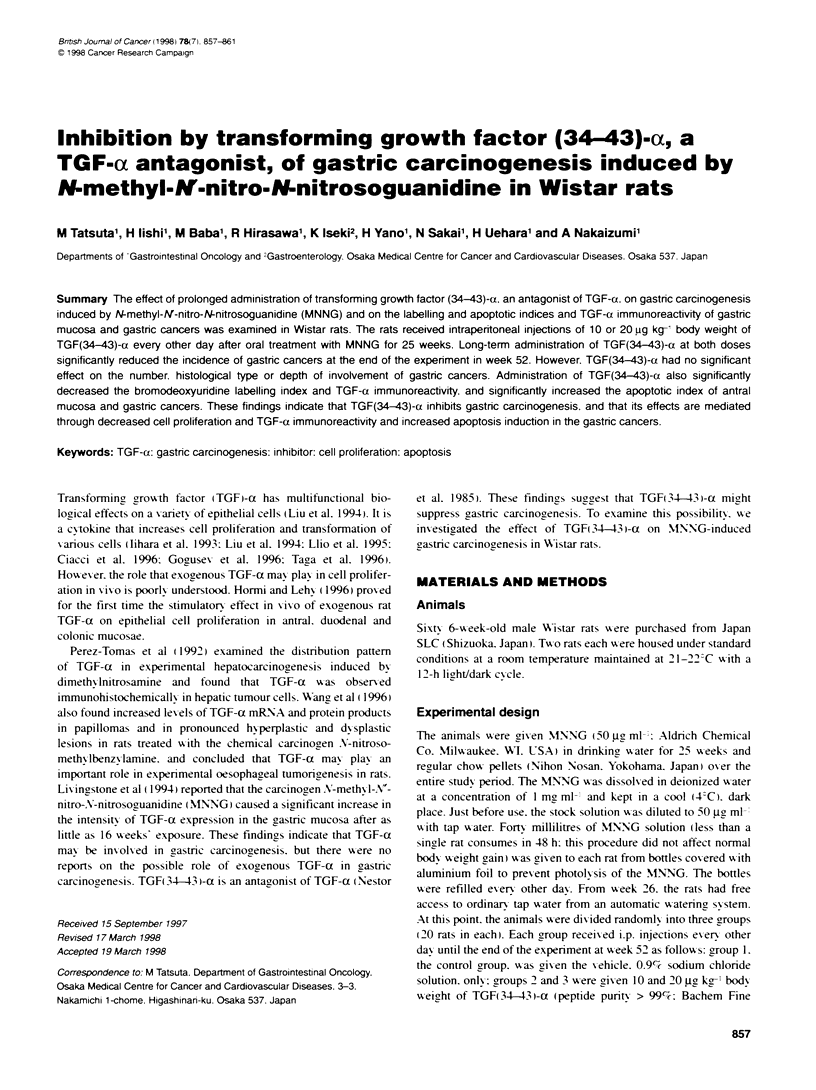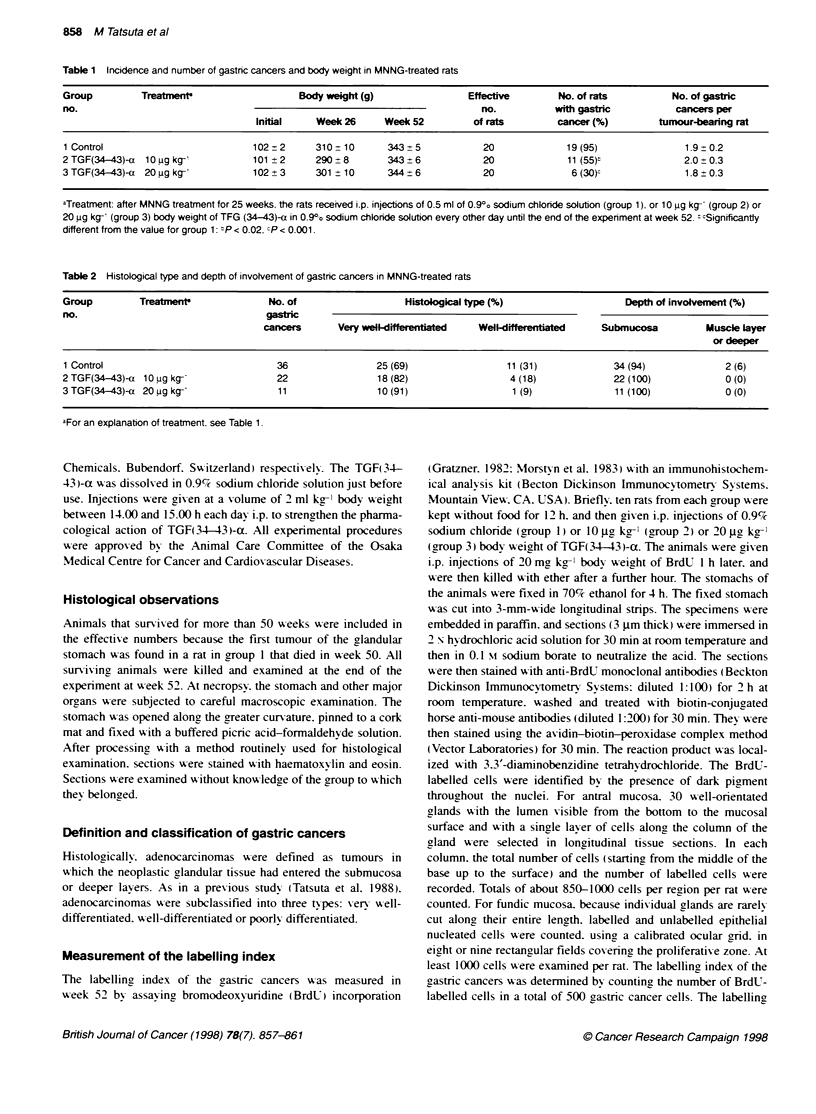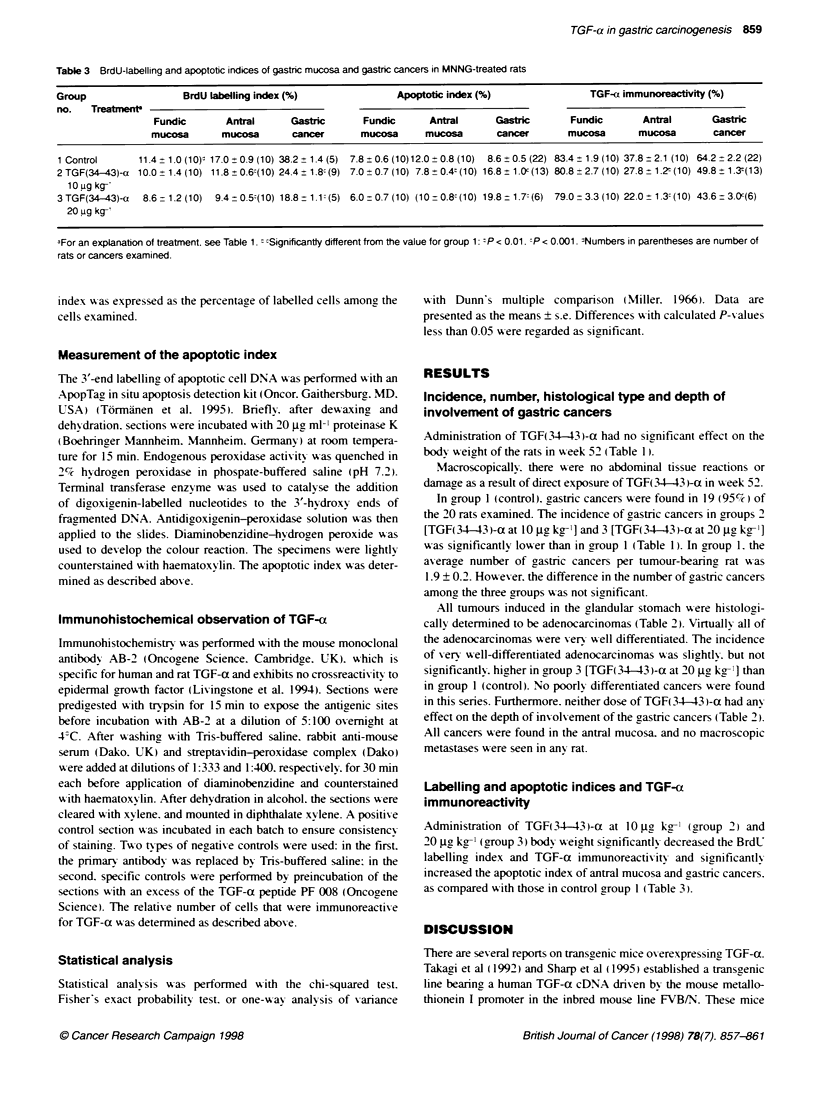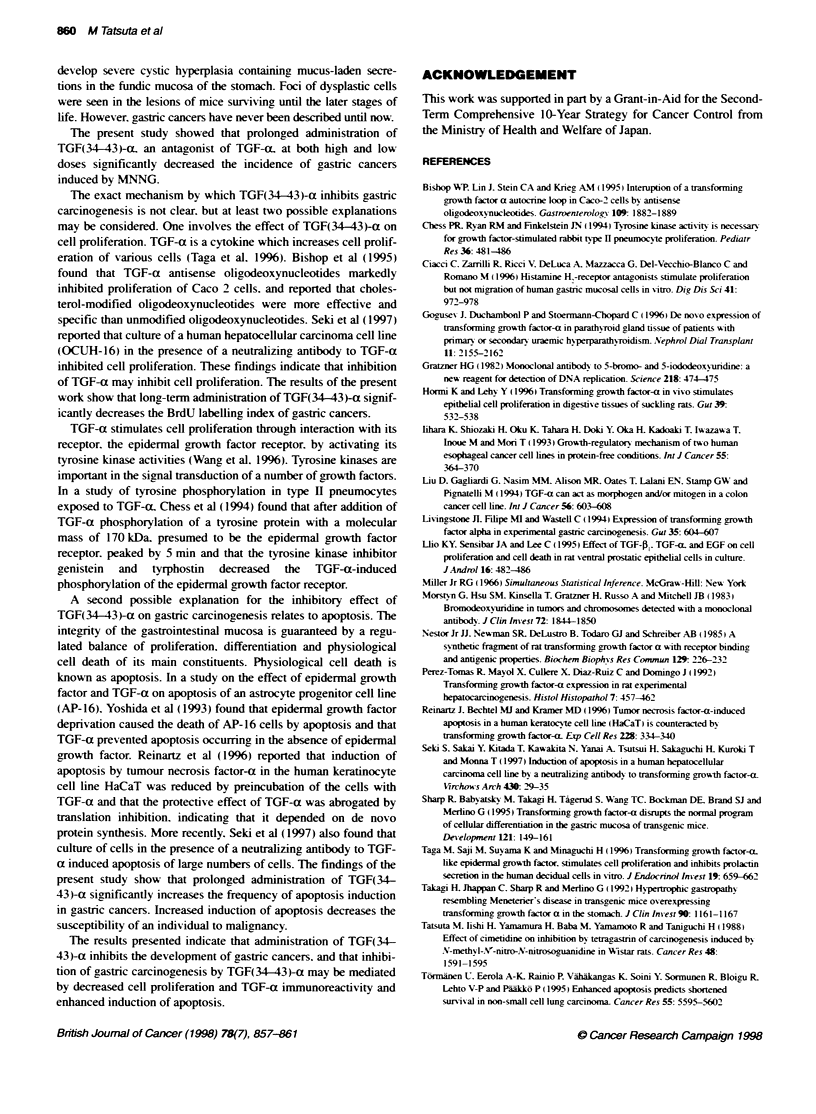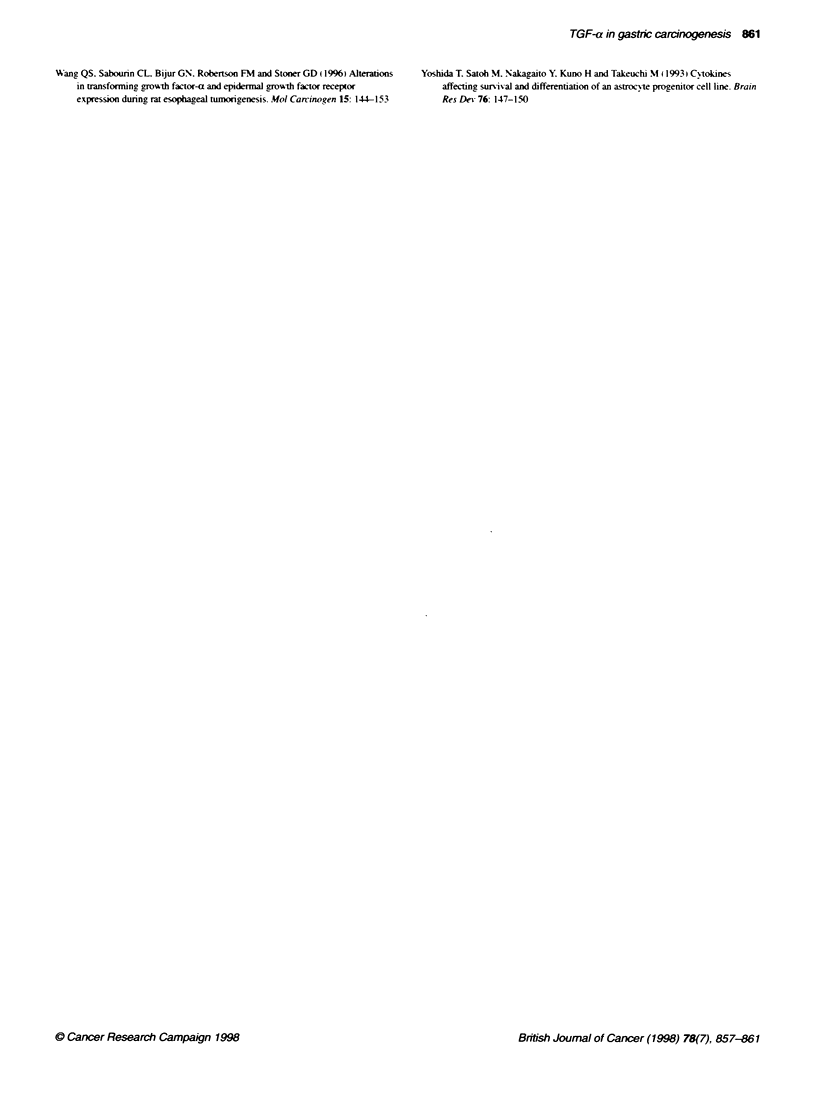# Inhibition by transforming growth factor (34-43)-alpha, a TGF-alpha antagonist, of gastric carcinogenesis induced by N-methyl-N'-nitro-N-nitrosoguanidine in Wistar rats.

**DOI:** 10.1038/bjc.1998.593

**Published:** 1998-10

**Authors:** M. Tatsuta, H. Iishi, M. Baba, R. Hirasawa, K. Iseki, H. Yano, N. Sakai, H. Uehara, A. Nakaizumi

**Affiliations:** Department of Gastrointestinal Oncology, Osaka Medical Centre for Cancer and Cardiovascular Diseases, Japan.

## Abstract

The effect of prolonged administration of transforming growth factor (34-43)-alpha, an antagonist of TGF-alpha, on gastric carcinogenesis induced by N-methyl-N'-nitro-N-nitrosoguanidine (MNNG) and on the labelling and apoptotic indices and TGF-alpha immunoreactivity of gastric mucosa and gastric cancers was examined in Wistar rats. The rats received intraperitoneal injections of 10 or 20 microg kg(-1) body weight of TGF(34-43)-alpha every other day after oral treatment with MNNG for 25 weeks. Long-term administration of TGF(34-43)-alpha at both doses significantly reduced the incidence of gastric cancers at the end of the experiment in week 52. However, TGF(34-43)-alpha had no significant effect on the number, histological type or depth of involvement of gastric cancers. Administration of TGF(34-43)-alpha also significantly decreased the bromodeoxyuridine labelling index and TGF-alpha immunoreactivity, and significantly increased the apoptotic index of antral mucosa and gastric cancers. These findings indicate that TGF(34-43)-alpha inhibits gastric carcinogenesis, and that its effects are mediated through decreased cell proliferation and TGF-alpha immunoreactivity and increased apoptosis induction in the gastric cancers.


British Journal of Cancer (1998) 78(7). 857–861
© 1998 Cancer Research Campaign

### Inhibition by transforming growth factor (34–43)-α, a TGF-α antagonist, of gastric carcinogenesis induced by *N*-methyl-*N'*-nitro-*N*-nitrosoguanidine in Wistar rats

**M Tatsuta[1], H Iishi[1], M Baba[1], R Hirasawa[1], K Iseki[2], H Yano[1], N Sakai[1], H Uehara[1] and A Nakaizumi[1]**

Departments of [1]Gastrointestinal Oncology and [2]Gastroenterology. Osaka Medical Centre for Cancer and Cardiovascular Diseases. Osaka 537. Japan

**Summary** The effect of prolonged administration of transforming growth factor (34–43)-α. an antagonist of TGF-α. on gastric carcinogenesis induced by *N*-methyl-*N'*-nitro-*N*-nitrosoguanidine (MNNG) and on the labelling and apoptotic indices and TGF-α immunoreactivity of gastric mucosa and gastric cancers was examined in Wistar rats. The rats received intraperitoneal injections of 10 or 20 μg $kg^{-1}$ body weight of TGF(34–43)-α every other day after oral treatment with MNNG for 25 weeks. Long-term administration of TGF(34–43)-α at both doses significantly reduced the incidence of gastric cancers at the end of the experiment in week 52. However. TGF(34–43)-α had no significant effect on the number. histological type or depth of involvement of gastric cancers. Administration of TGF(34–43)-α also significantly decreased the bromodeoxyuridine labelling index and TGF-α immunoreactivity. and significantly increased the apoptotic index of antral mucosa and gastric cancers. These findings indicate that TGF(34–43)-α inhibits gastric carcinogenesis. and that its effects are mediated through decreased cell proliferation and TGF-α immunoreactivity and increased apoptosis induction in the gastric cancers.

**Keywords:** TGF-α: gastric carcinogenesis: inhibitor: cell proliferation: apoptosis

Transforming growth factor (TGF)-α has multifunctional biological effects on a variety of epithelial cells (Liu et al. 1994). It is a cytokine that increases cell proliferation and transformation of various cells (Iihara et al. 1993: Liu et al. 1994: Llio et al. 1995: Ciacci et al. 1996: Gogusev et al. 1996: Taga et al. 1996). However. the role that exogenous TGF-α may play in cell proliferation in vivo is poorly understood. Hormi and Lehy (1996) proved for the first time the stimulatory effect in vivo of exogenous rat TGF-α on epithelial cell proliferation in antral. duodenal and colonic mucosae.

Perez-Tomas et al (1992) examined the distribution pattern of TGF-α in experimental hepatocarcinogenesis induced by dimethylnitrosamine and found that TGF-α was observed immunohistochemically in hepatic tumour cells. Wang et al (1996) also found increased levels of TGF-α mRNA and protein products in papillomas and in pronounced hyperplastic and dysplastic lesions in rats treated with the chemical carcinogen N-nitrosomethylbenzylamine. and concluded that TGF-α may play an important role in experimental oesophageal tumorigenesis in rats. Livingstone et al (1994) reported that the carcinogen N-methyl-N'-nitro-N-nitrosoguanidine (MNNG) caused a significant increase in the intensity of TGF-α expression in the gastric mucosa after as little as 16 weeks' exposure. These findings indicate that TGF-α may be involved in gastric carcinogenesis. but there were no reports on the possible role of exogenous TGF-α in gastric carcinogenesis. TGF(34–43)-α is an antagonist of TGF-α (Nestor et al. 1985). These findings suggest that TGF(34–43)-α might suppress gastric carcinogenesis. To examine this possibility. we investigated the effect of TGF(34–43)-α on MNNG-induced gastric carcinogenesis in Wistar rats.

*Received 15 September 1997*
*Revised 17 March 1998*
*Accepted 19 March 1998*

*Correspondence to:* M Tatsuta. Department of Gastrointestinal Oncology. Osaka Medical Centre for Cancer and Cardiovascular Diseases. 3–3. Nakamichi 1-chome. Higashinari-ku. Osaka 537. Japan

### MATERIALS AND METHODS

### Animals

Sixty 6-week-old male Wistar rats were purchased from Japan SLC (Shizuoka. Japan). Two rats each were housed under standard conditions at a room temperature maintained at 21–22°C with a 12-h light/dark cycle.

### Experimental design

The animals were given MNNG (50 μg $ml^{-1}$: Aldrich Chemical Co. Milwaukee. WI. USA) in drinking water for 25 weeks and regular chow pellets (Nihon Nosan. Yokohama. Japan) over the entire study period. The MNNG was dissolved in deionized water at a concentration of 1 mg $ml^{-1}$ and kept in a cool (4°C). dark place. Just before use. the stock solution was diluted to 50 μg $ml^{-1}$ with tap water. Forty millilitres of MNNG solution (less than a single rat consumes in 48 h: this procedure did not affect normal body weight gain) was given to each rat from bottles covered with aluminium foil to prevent photolysis of the MNNG. The bottles were refilled every other day. From week 26. the rats had free access to ordinary tap water from an automatic watering system. At this point. the animals were divided randomly into three groups (20 rats in each). Each group received i.p. injections every other day until the end of the experiment at week 52 as follows: group 1. the control group. was given the vehicle. 0.9% sodium chloride solution. only: groups 2 and 3 were given 10 and 20 μg $kg^{-1}$ body weight of TGF(34–43)-α (peptide purity > 99%: Bachem Fine

Table 1 Incidence and number of gastric cancers and body weight in MNNG-treated rats

| Group no. | Treatment[a] | Body weight (g) | | | Effective no. of rats | No. of rats with gastric cancer (%) | No. of gastric cancers per tumour-bearing rat |
|---|---|---|---|---|---|---|---|
| | | Initial | Week 26 | Week 52 | | | |
| 1 Control | | 102 ± 2 | 310 ± 10 | 343 ± 5 | 20 | 19 (95) | 1.9 ± 0.2 |
| 2 TGF(34–43)-α | 10 μg kg⁻¹ | 101 ± 2 | 290 ± 8 | 343 ± 6 | 20 | 11 (55)[b] | 2.0 ± 0.3 |
| 3 TGF(34–43)-α | 20 μg kg⁻¹ | 102 ± 3 | 301 ± 10 | 344 ± 6 | 20 | 6 (30)[c] | 1.8 ± 0.3 |

[a]Treatment: after MNNG treatment for 25 weeks, the rats received i.p. injections of 0.5 ml of 0.9% sodium chloride solution (group 1), or 10 μg $kg^{-1}$ (group 2) or 20 μg $kg^{-1}$ (group 3) body weight of TFG (34–43)-α in 0.9% sodium chloride solution every other day until the end of the experiment at week 52. [b,c]Significantly different from the value for group 1: [b]$P < 0.02$, [c]$P < 0.001$.

Table 2 Histological type and depth of involvement of gastric cancers in MNNG-treated rats

| Group no. | Treatment[a] | No. of gastric cancers | Histological type (%) | | Depth of involvement (%) | |
|---|---|---|---|---|---|---|
| | | | Very well-differentiated | Well-differentiated | Submucosa | Muscle layer or deeper |
| 1 Control | | 36 | 25 (69) | 11 (31) | 34 (94) | 2 (6) |
| 2 TGF(34–43)-α | 10 μg kg⁻¹ | 22 | 18 (82) | 4 (18) | 22 (100) | 0 (0) |
| 3 TGF(34–43)-α | 20 μg kg⁻¹ | 11 | 10 (91) | 1 (9) | 11 (100) | 0 (0) |

[a]For an explanation of treatment, see Table 1.

Chemicals, Bubendorf, Switzerland) respectively. The TGF(34–43)-α was dissolved in 0.9% sodium chloride solution just before use. Injections were given at a volume of 2 ml $kg^{-1}$ body weight between 14.00 and 15.00 h each day i.p. to strengthen the pharmacological action of TGF(34–43)-α. All experimental procedures were approved by the Animal Care Committee of the Osaka Medical Centre for Cancer and Cardiovascular Diseases.

### Histological observations

Animals that survived for more than 50 weeks were included in the effective numbers because the first tumour of the glandular stomach was found in a rat in group 1 that died in week 50. All surviving animals were killed and examined at the end of the experiment at week 52. At necropsy, the stomach and other major organs were subjected to careful macroscopic examination. The stomach was opened along the greater curvature, pinned to a cork mat and fixed with a buffered picric acid–formaldehyde solution. After processing with a method routinely used for histological examination, sections were stained with haematoxylin and eosin. Sections were examined without knowledge of the group to which they belonged.

### Definition and classification of gastric cancers

Histologically, adenocarcinomas were defined as tumours in which the neoplastic glandular tissue had entered the submucosa or deeper layers. As in a previous study (Tatsuta et al, 1988), adenocarcinomas were subclassified into three types: very well-differentiated, well-differentiated or poorly differentiated.

### Measurement of the labelling index

The labelling index of the gastric cancers was measured in week 52 by assaying bromodeoxyuridine (BrdU) incorporation (Gratzner, 1982; Morstyn et al, 1983) with an immunohistochemical analysis kit (Becton Dickinson Immunocytometry Systems, Mountain View, CA, USA). Briefly, ten rats from each group were kept without food for 12 h, and then given i.p. injections of 0.9% sodium chloride (group 1) or 10 μg $kg^{-1}$ (group 2) or 20 μg $kg^{-1}$ (group 3) body weight of TGF(34–43)-α. The animals were given i.p. injections of 20 mg $kg^{-1}$ body weight of BrdU 1 h later, and were then killed with ether after a further hour. The stomachs of the animals were fixed in 70% ethanol for 4 h. The fixed stomach was cut into 3-mm-wide longitudinal strips. The specimens were embedded in paraffin, and sections (3 μm thick) were immersed in 2 N hydrochloric acid solution for 30 min at room temperature and then in 0.1 M sodium borate to neutralize the acid. The sections were then stained with anti-BrdU monoclonal antibodies (Beckton Dickinson Immunocytometry Systems; diluted 1:100) for 2 h at room temperature, washed and treated with biotin-conjugated horse anti-mouse antibodies (diluted 1:200) for 30 min. They were then stained using the avidin–biotin–peroxidase complex method (Vector Laboratories) for 30 min. The reaction product was localized with 3,3′-diaminobenzidine tetrahydrochloride. The BrdU-labelled cells were identified by the presence of dark pigment throughout the nuclei. For antral mucosa, 30 well-orientated glands with the lumen visible from the bottom to the mucosal surface and with a single layer of cells along the column of the gland were selected in longitudinal tissue sections. In each column, the total number of cells (starting from the middle of the base up to the surface) and the number of labelled cells were recorded. Totals of about 850–1000 cells per region per rat were counted. For fundic mucosa, because individual glands are rarely cut along their entire length, labelled and unlabelled epithelial nucleated cells were counted, using a calibrated ocular grid, in eight or nine rectangular fields covering the proliferative zone. At least 1000 cells were examined per rat. The labelling index of the gastric cancers was determined by counting the number of BrdU-labelled cells in a total of 500 gastric cancer cells. The labelling

© Cancer Research Campaign 1998

**Table 3** BrdU-labelling and apoptotic indices of gastric mucosa and gastric cancers in MNNG-treated rats

| Group no. | Treatment[a] | BrdU labelling index (%) | | | Apoptotic index (%) | | | TGF-α immunoreactivity (%) | | |
|---|---|---|---|---|---|---|---|---|---|---|
| | | Fundic mucosa | Antral mucosa | Gastric cancer | Fundic mucosa | Antral mucosa | Gastric cancer | Fundic mucosa | Antral mucosa | Gastric cancer |
| 1 | Control | 11.4 ± 1.0 (10)[d] | 17.0 ± 0.9 (10) | 38.2 ± 1.4 (5) | 7.8 ± 0.6 (10) | 12.0 ± 0.8 (10) | 8.6 ± 0.5 (22) | 83.4 ± 1.9 (10) | 37.8 ± 2.1 (10) | 64.2 ± 2.2 (22) |
| 2 | TGF(34–43)-α 10 μg kg$^{-1}$ | 10.0 ± 1.4 (10) | 11.8 ± 0.6[c] (10) | 24.4 ± 1.8[c] (9) | 7.0 ± 0.7 (10) | 7.8 ± 0.4[b] (10) | 16.8 ± 1.0[c] (13) | 80.8 ± 2.7 (10) | 27.8 ± 1.2[c] (10) | 49.8 ± 1.3[c] (13) |
| 3 | TGF(34–43)-α 20 μg kg$^{-1}$ | 8.6 ± 1.2 (10) | 9.4 ± 0.5[c] (10) | 18.8 ± 1.1[c] (5) | 6.0 ± 0.7 (10) | (10 ± 0.8[c] (10) | 19.8 ± 1.7[c] (6) | 79.0 ± 3.3 (10) | 22.0 ± 1.3[c] (10) | 43.6 ± 3.0[c] (6) |

[a]For an explanation of treatment, see Table 1. [b,c]Significantly different from the value for group 1: [b]$P < 0.01$. [c]$P < 0.001$. [d]Numbers in parentheses are number of rats or cancers examined.

index was expressed as the percentage of labelled cells among the cells examined.

### Measurement of the apoptotic index

The 3′-end labelling of apoptotic cell DNA was performed with an ApopTag in situ apoptosis detection kit (Oncor, Gaithersburg, MD, USA) (Törmänen et al, 1995). Briefly, after dewaxing and dehydration, sections were incubated with 20 μg ml$^{-1}$ proteinase K (Boehringer Mannheim, Mannheim, Germany) at room temperature for 15 min. Endogenous peroxidase activity was quenched in 2% hydrogen peroxidase in phospate-buffered saline (pH 7.2). Terminal transferase enzyme was used to catalyse the addition of digoxigenin-labelled nucleotides to the 3′-hydroxy ends of fragmented DNA. Antidigoxigenin–peroxidase solution was then applied to the slides. Diaminobenzidine–hydrogen peroxide was used to develop the colour reaction. The specimens were lightly counterstained with haematoxylin. The apoptotic index was determined as described above.

### Immunohistochemical observation of TGF-α

Immunohistochemistry was performed with the mouse monoclonal antibody AB-2 (Oncogene Science, Cambridge, UK), which is specific for human and rat TGF-α and exhibits no crossreactivity to epidermal growth factor (Livingstone et al, 1994). Sections were predigested with trypsin for 15 min to expose the antigenic sites before incubation with AB-2 at a dilution of 5:100 overnight at 4°C. After washing with Tris-buffered saline, rabbit anti-mouse serum (Dako, UK) and streptavidin–peroxidase complex (Dako) were added at dilutions of 1:333 and 1:400, respectively, for 30 min each before application of diaminobenzidine and counterstained with haematoxylin. After dehydration in alcohol, the sections were cleared with xylene, and mounted in diphthalate xylene. A positive control section was incubated in each batch to ensure consistency of staining. Two types of negative controls were used: in the first, the primary antibody was replaced by Tris-buffered saline; in the second, specific controls were performed by preincubation of the sections with an excess of the TGF-α peptide PF 008 (Oncogene Science). The relative number of cells that were immunoreactive for TGF-α was determined as described above.

### Statistical analysis

Statistical analysis was performed with the chi-squared test, Fisher's exact probability test, or one-way analysis of variance with Dunn's multiple comparison (Miller, 1966). Data are presented as the means ± s.e. Differences with calculated $P$-values less than 0.05 were regarded as significant.

### RESULTS

### Incidence, number, histological type and depth of involvement of gastric cancers

Administration of TGF(34–43)-α had no significant effect on the body weight of the rats in week 52 (Table 1).

Macroscopically, there were no abdominal tissue reactions or damage as a result of direct exposure of TGF(34–43)-α in week 52.

In group 1 (control), gastric cancers were found in 19 (95%) of the 20 rats examined. The incidence of gastric cancers in groups 2 [TGF(34–43)-α at 10 μg kg$^{-1}$] and 3 [TGF(34–43)-α at 20 μg kg$^{-1}$] was significantly lower than in group 1 (Table 1). In group 1, the average number of gastric cancers per tumour-bearing rat was 1.9 ± 0.2. However, the difference in the number of gastric cancers among the three groups was not significant.

All tumours induced in the glandular stomach were histologically determined to be adenocarcinomas (Table 2). Virtually all of the adenocarcinomas were very well differentiated. The incidence of very well-differentiated adenocarcinomas was slightly, but not significantly, higher in group 3 [TGF(34–43)-α at 20 μg kg$^{-1}$] than in group 1 (control). No poorly differentiated cancers were found in this series. Furthermore, neither dose of TGF(34–43)-α had any effect on the depth of involvement of the gastric cancers (Table 2). All cancers were found in the antral mucosa, and no macroscopic metastases were seen in any rat.

### Labelling and apoptotic indices and TGF-α immunoreactivity

Administration of TGF(34–43)-α at 10 μg kg$^{-1}$ (group 2) and 20 μg kg$^{-1}$ (group 3) body weight significantly decreased the BrdU labelling index and TGF-α immunoreactivity and significantly increased the apoptotic index of antral mucosa and gastric cancers, as compared with those in control group 1 (Table 3).

### DISCUSSION

There are several reports on transgenic mice overexpressing TGF-α. Takagi et al (1992) and Sharp et al (1995) established a transgenic line bearing a human TGF-α cDNA driven by the mouse metallothionein I promoter in the inbred mouse line FVB/N. These mice

© Cancer Research Campaign 1998

develop severe cystic hyperplasia containing mucus-laden secretions in the fundic mucosa of the stomach. Foci of dysplastic cells were seen in the lesions of mice surviving until the later stages of life. However, gastric cancers have never been described until now.

The present study showed that prolonged administration of TGF(34–43)-α, an antagonist of TGF-α, at both high and low doses significantly decreased the incidence of gastric cancers induced by MNNG.

The exact mechanism by which TGF(34–43)-α inhibits gastric carcinogenesis is not clear, but at least two possible explanations may be considered. One involves the effect of TGF(34–43)-α on cell proliferation. TGF-α is a cytokine which increases cell proliferation of various cells (Taga et al, 1996). Bishop et al (1995) found that TGF-α antisense oligodeoxynucleotides markedly inhibited proliferation of Caco 2 cells, and reported that cholesterol-modified oligodeoxynucleotides were more effective and specific than unmodified oligodeoxynucleotides. Seki et al (1997) reported that culture of a human hepatocellular carcinoma cell line (OCUH-16) in the presence of a neutralizing antibody to TGF-α inhibited cell proliferation. These findings indicate that inhibition of TGF-α may inhibit cell proliferation. The results of the present work show that long-term administration of TGF(34–43)-α significantly decreases the BrdU labelling index of gastric cancers.

TGF-α stimulates cell proliferation through interaction with its receptor, the epidermal growth factor receptor, by activating its tyrosine kinase activities (Wang et al, 1996). Tyrosine kinases are important in the signal transduction of a number of growth factors. In a study of tyrosine phosphorylation in type II pneumocytes exposed to TGF-α, Chess et al (1994) found that after addition of TGF-α phosphorylation of a tyrosine protein with a molecular mass of 170 kDa, presumed to be the epidermal growth factor receptor, peaked by 5 min and that the tyrosine kinase inhibitor genistein and tyrphostin decreased the TGF-α-induced phosphorylation of the epidermal growth factor receptor.

A second possible explanation for the inhibitory effect of TGF(34–43)-α on gastric carcinogenesis relates to apoptosis. The integrity of the gastrointestinal mucosa is guaranteed by a regulated balance of proliferation, differentiation and physiological cell death of its main constituents. Physiological cell death is known as apoptosis. In a study on the effect of epidermal growth factor and TGF-α on apoptosis of an astrocyte progenitor cell line (AP-16), Yoshida et al (1993) found that epidermal growth factor deprivation caused the death of AP-16 cells by apoptosis and that TGF-α prevented apoptosis occurring in the absence of epidermal growth factor. Reinartz et al (1996) reported that induction of apoptosis by tumour necrosis factor-α in the human keratinocyte cell line HaCaT was reduced by preincubation of the cells with TGF-α and that the protective effect of TGF-α was abrogated by translation inhibition, indicating that it depended on de novo protein synthesis. More recently, Seki et al (1997) also found that culture of cells in the presence of a neutralizing antibody to TGF-α induced apoptosis of large numbers of cells. The findings of the present study show that prolonged administration of TGF(34–43)-α significantly increases the frequency of apoptosis induction in gastric cancers. Increased induction of apoptosis decreases the susceptibility of an individual to malignancy.

The results presented indicate that administration of TGF(34–43)-α inhibits the development of gastric cancers, and that inhibition of gastric carcinogenesis by TGF(34–43)-α may be mediated by decreased cell proliferation and TGF-α immunoreactivity and enhanced induction of apoptosis.

### ACKNOWLEDGEMENT

This work was supported in part by a Grant-in-Aid for the Second-Term Comprehensive 10-Year Strategy for Cancer Control from the Ministry of Health and Welfare of Japan.

### REFERENCES

Bishop WP, Lin J, Stein CA and Krieg AM (1995) Interuption of a transforming growth factor α autocrine loop in Caco-2 cells by antisense oligodeoxynucleotides. *Gastroenterology* **109**: 1882–1889

Chess PR, Ryan RM and Finkelstein JN (1994) Tyrosine kinase activity is necessary for growth factor-stimulated rabbit type II pneumocyte proliferation. *Pediatr Res* **36**: 481–486

Ciacci C, Zarrilli R, Ricci V, DeLuca A, Mazzacca G, Del-Vecchio-Blanco C and Romano M (1996) Histamine $H_2$-receptor antagonists stimulate proliferation but not migration of human gastric mucosal cells in vitro. *Dig Dis Sci* **41**: 972–978

Gogusev J, Duchambonl P and Stoermann-Chopard C (1996) De novo expression of transforming growth factor-α in parathyroid gland tissue of patients with primary or secondary uraemic hyperparathyroidism. *Nephrol Dial Transplant* **11**: 2155–2162

Gratzner HG (1982) Monoclonal antibody to 5-bromo- and 5-iododeoxyuridine: a new reagent for detection of DNA replication. *Science* **218**: 474–475

Hormi K and Lehy Y (1996) Transforming growth factor-α in vivo stimulates epithelial cell proliferation in digestive tissues of suckling rats. *Gut* **39**: 532–538

Iihara K, Shiozaki H, Oku K, Tahara H, Doki Y, Oka H, Kadoaki T, Iwazawa T, Inoue M and Mori T (1993) Growth-regulatory mechanism of two human esophageal cancer cell lines in protein-free conditions. *Int J Cancer* **55**: 364–370

Liu D, Gagliardi G, Nasim MM, Alison MR, Oates T, Lalani EN, Stamp GW and Pignatelli M (1994) TGF-α can act as morphogen and/or mitogen in a colon cancer cell line. *Int J Cancer* **56**: 603–608

Livingstone JI, Filipe MI and Wastell C (1994) Expression of transforming growth factor alpha in experimental gastric carcinogenesis. *Gut* **35**: 604–607

Llio KY, Sensibar JA and Lee C (1995) Effect of TGF-$\beta_1$, TGF-α, and EGF on cell proliferation and cell death in rat ventral prostatic epithelial cells in culture. *J Androl* **16**: 482–486

Miller Jr RG (1966) *Simultaneous Statistical Inference*. McGraw-Hill: New York

Morstyn G, Hsu SM, Kinsella T, Gratzner H, Russo A and Mitchell JB (1983) Bromodeoxyuridine in tumors and chromosomes detected with a monoclonal antibody. *J Clin Invest* **72**: 1844–1850

Nestor Jr JJ, Newman SR, DeLustro B, Todaro GJ and Schreiber AB (1985) A synthetic fragment of rat transforming growth factor α with receptor binding and antigenic properties. *Biochem Biophys Res Commun* **129**: 226–232

Perez-Tomas R, Mayol X, Cullere X, Diaz-Ruiz C and Domingo J (1992) Transforming growth factor-α expression in rat experimental hepatocarcinogenesis. *Histol Histopathol* **7**: 457–462

Reinartz J, Bechtel MJ and Kramer MD (1996) Tumor necrosis factor-α-induced apoptosis in a human keratocyte cell line (HaCaT) is counteracted by transforming growth factor-α. *Exp Cell Res* **228**: 334–340

Seki S, Sakai Y, Kitada T, Kawakita N, Yanai A, Tsutsui H, Sakaguchi H, Kuroki T and Monna T (1997) Induction of apoptosis in a human hepatocellular carcinoma cell line by a neutralizing antibody to transforming growth factor-α. *Virchows Arch* **430**: 29–35

Sharp R, Babyatsky M, Takagi H, Tågerud S, Wang TC, Bockman DE, Brand SJ and Merlino G (1995) Transforming growth factor-α disrupts the normal program of cellular differentiation in the gastric mucosa of transgenic mice. *Development* **121**: 149–161

Taga M, Saji M, Suyama K and Minaguchi H (1996) Transforming growth factor-α-like epidermal growth factor, stimulates cell proliferation and inhibits prolactin secretion in the human decidual cells in vitro. *J Endocrinol Invest* **19**: 659–662

Takagi H, Jhappan C, Sharp R and Merlino G (1992) Hypertrophic gastropathy resembling Meneterier's disease in transgenic mice overexpressing transforming growth factor α in the stomach. *J Clin Invest* **90**: 1161–1167

Tatsuta M, Iishi H, Yamamura H, Baba M, Yamamoto R and Taniguchi H (1988) Effect of cimetidine on inhibition by tetragastrin of carcinogenesis induced by *N*-methyl-*N'*-nitro-*N*-nitrosoguanidine in Wistar rats. *Cancer Res* **48**: 1591–1595

Törmänen U, Eerola A-K, Rainio P, Vähäkangas K, Soini Y, Sormunen R, Bloigu R, Lehto V-P and Pääkkö P (1995) Enhanced apoptosis predicts shortened survival in non-small cell lung carcinoma. *Cancer Res* **55**: 5595–5602

© Cancer Research Campaign 1998

Wang QS, Sabourin CL, Bijur GN, Robertson FM and Stoner GD (1996) Alterations in transforming growth factor-α and epidermal growth factor receptor expression during rat esophageal tumorigenesis. *Mol Carcinogen* **15**: 144–153

Yoshida T, Satoh M, Nakagaito Y, Kuno H and Takeuchi M (1993) Cytokines affecting survival and differentiation of an astrocyte progenitor cell line. *Brain Res Dev* **76**: 147–150

*© Cancer Research Campaign 1998*